# An Alternative Micro-Milling Fabrication Process for Rapid and Low-Cost Microfluidics

**DOI:** 10.3390/mi15070905

**Published:** 2024-07-11

**Authors:** Martin Christopher Allen, Simon Lookmire, Ebubekir Avci

**Affiliations:** 1 College of Sciences, School of Food and Advanced Technology, Massey University, Palmerston North 4410, New Zealand; S.Lookmire@massey.ac.nz; 2 The MacDiarmid Institute, Victoria University of Wellington, P.O. Box 600, Wellington 6140, New Zealand; E.Avci@massey.ac.nz

**Keywords:** microfluidics, microfabrication, micro-CNC milling, CNC, microparticle manipulation, profilometry, image processing

## Abstract

Microfluidics is an important technology for the biomedical industry and is often utilised in our daily lives. Recent advances in micro-milling technology have allowed for rapid fabrication of smaller and more complex structures, at lower costs, making it a viable alternative to other fabrication methods. The microfluidic chip fabrication developed in this research is a step-by-step process with a self-contained wet milling chamber. Additionally, ethanol solvent bonding is used to allow microfluidic chips to be fully fabricated within approximately an hour. The effect of using this process is tested with quantitative contact profileometery data to determine the expected surface roughness in the microchannels. The effect of surface roughness on the controllability of microparticles is tested in functional microfluidic chips using image processing to calculate particle velocity. This process can produce high-quality channels when compared with similar studies in the literature and surface roughness affects the control of microparticles. Lastly, we discuss how the outcomes of this research can produce rapid and higher-quality microfluidic devices, leading to improvement in the research and development process within the fields of science that utilise microfluidic technology. Such as medicine, biology, chemistry, ecology, and aerospace.

## 1. Introduction

Microfluidics is the study of the behaviour, precise control, and manipulation of fluids that are too small to be seen by the naked eye, typically at a microscale [1,2,3]. Microfluidics has been extensively commercialised since the 1970s in inkjet printing [4,5]. As the technology evolved with the development of new fabrication methods for microfluidics, e.g., photolithography [6,7], soft lithography [8,9], and advanced 3D printing methods [10,11], combined with access to low-cost polymer fabrication [12], microfluidics has become significantly more accessible to researchers and industries.

Photolithography is a standard process used for fabricating microfluidic chips and has been inherited from microelectronic industries. Channels made using this process have high spatial resolutions, along with extremely smooth surfaces, and can be processed in parallel [6,7]. However, equipment, maintenance, and facilities required to fabricate in this way are expensive and time-consuming to configure. Moreover, features cannot be complex and are fixed to 2.5D [13,14].

Soft lithography (otherwise referred to as PDMS casting) is an established method for producing chips at a lower cost. By using a subtractive process like micro-milling or photolithography a mould can be created [9,11,15]. The PDMS polymer replicates the surface features and can form a water-tight seal against itself, glass or any smooth surface [13,16]. However, it is susceptible to distortions and defects [9] and requires additional manual processing steps making it difficult to automate [15,17].

Three-dimensional (3D) printing is suited for rapid manufacturing, allowing for near-full automation of the process. Internal structures can be more complex and functionalities can be assessed digitally. However, there is a limitation to the selection of bio-compatible resins that may have restricted proprietary information, reducing versatility. Other physical limitations include lower spatial resolutions and throughput compared to other conventional processes [10,17].

Recent advances in micro-milling technology, including reducing tools down to 5 µm, as well as more available diverse tool sets, allow for complex fabrication [18], making micro-milling an attractive alternative to other fabrication methods. Micro-milling can create more complex structures like completely circular microchannels [15]; modular microfluidic systems in injection-moulded bricks [19]; or channels with varying sizes, lengths, and depths [20]. Unlike 3D printing, micro-milling is not restricted to certain polymers and can work on many different plastics and metals [21,22]. Additional axes (beyond the 3-axis) also allow for more complex operations, e.g., undercuts. Moreover, low capital and running costs, low level of expertise required, and short processing times, make micro-milling ideal for rapid prototyping of microfluidic systems [21,23]. The estimated costs per chip (once factoring in materials and machine usage) are approximately USD 1 to USD 6 [21,24].

In our previous research [25], the optimal milling parameters were investigated for 100 µm tools and the high variance in milling parameters and surface roughness (ranging from 420 nm to 24 nm) are expressed in [21,26,27,28]. Additionally, it was determined that the optimal parameters for PMMA (poly(methyl methacrylate)) are 60,000 RPM at a 200 mm/min feed rate, which results in the smoothest surface. Building on this prior work [25] the current study calculates a roughness value based on quantitative data, allowing for direct comparison with the literature. Additionally, the use of wet milling is explored to enhance the functionality of microfluidic chips. The entire fabrication process was examined by assessing the functionality of complete microfluidic chip systems and determining how surface quality impacts the controllability of these chips. The novel aspects of this paper include the following:

1. A unique step-by-step wet fabrication method for micro-milling machines that is self-contained and attaches to existing systems using universal fasteners and low-cost materials to produce functional microfluidic chips. Alternatives involved blasting milling fluid at the tool [29], requiring additional equipment and reducing tool life, or placing water droplets [28], which is insufficient for larger operations and can cause corrosion.
2. Demonstration of the advantage of using smooth fabricated surfaces over rough ones by producing and testing a microparticle sorting chip. Operated using a time-efficient, low-cost microfluidic pumping and attachment system. Other experiments in the literature examined the functionality of their microfluidic chips, but without observing the difference between rough and smooth microchannels on microfluidic chip performance [21,26,27,28].

This paper describes the complete fabrication process used to create microfluidic chips (Section 2), involving material selection, how micro-milling is conducted, which solvent is used, and the process to enclose the chips. Two kinds of chips are fabricated, one for collecting contact profilometry data in an experimental design with different milling parameters and using both a wet and dry process. The other is for physically interacting with microparticles to experimentally demonstrate the practical applicability of our process and the differences between using chips with rough and smooth surfaces. The results from these experiments are analysed and discussed in relation to the physical appearance (Section 3.1), surface roughness (Section 3.2), observed particle behaviour (Section 3.3), and the potential impacts on the field of microfluidics (Section 3.4). Lastly, the advances and limitations of this research will be presented in Section 4).

## 2. Materials and Methods

**Figure 1:**
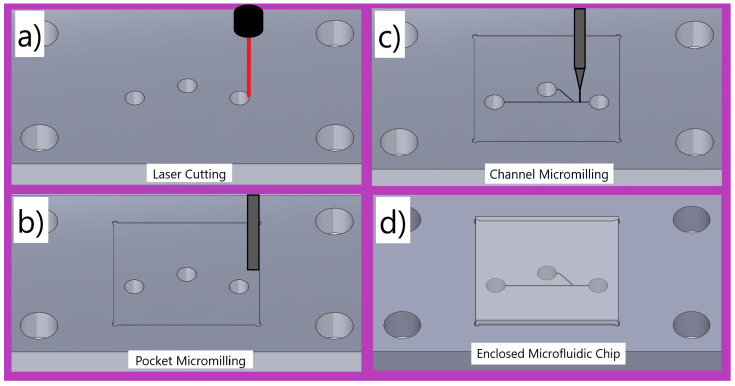
The complete fabrication process from acrylic sheet to functional microfluidic chip. (**a**) A laser cutter is used to cut the inlet/outlet holes, the corner fastening holes, and the body of the chip from the sheet of material. This is also used to cut out the enclosing material. (**b**) A 500 µm end mill is used to machine out the 200 µm deep pocket (recessed area). (**c**) A 100 µm end mill is used to machine the microchannels. (**d**) The channel is enclosed with a 3 mm thick piece of PMMA, which is milled down to 500 µm with a 1 mm end mill.

The main fabrication process used to create fully functional microfluidic chips is demonstrated in Figure 1. In Figure 1a, a laser cutter (Fusion Pro 48, Epilog Laser, Golden, CO, USA) is used to cut the inlet/outlet holes, the corner fastening holes, and the body of the chip from the sheet of material. It is also used to cut out the enclosing material. In Figure 1b, a 500 µm end mill is used to machine out the pocket, and then in Figure 1c, a 100 µm end mill is used to machine the microchannels. In Figure 1d, the channel is enclosed with a 3 mm thick piece of PMMA, which is milled down to 500 µm with a 1 mm end mill.

### 2.1. Materials

There is a diverse range of materials that can be utilised for fabricating microfluidic chips [30]. When compared with the set of materials suitable for micro-milling [21,22], the two sets converge on thermoplastics. Thermoplastics are among the most cost-effective materials for microfluidics, offering versatility for various applications ranging from chemistry to bio-culture [30]. PMMA is a desirable thermoplastic to use due to its high degree of optical transparency, bio-compatibility, non-porosity, and durability [31]. It is also affordable and easy to source. The technical data sheet for the PMMA sheet is provided in the Supplementary Materials document.

### 2.2. Chip Designs

Two different chip designs were used to test the micro-milling process. Both chips have large 6.5 mm holes for M6 screws to be directly fixed to an inch x inch grid of holes. The chips were designed in an AutoCAD program called SOLIDWORKS (SOLIDWORKS Education Edition Academic Year 2021-2022/2021 SP5.1, Dassault Systèmes, Vélizy-Villacoublay, France), allowing for easy modification of designs. The tool paths for micro-milling the microfluidic chips were also programmed in this software.

**Figure 2:**
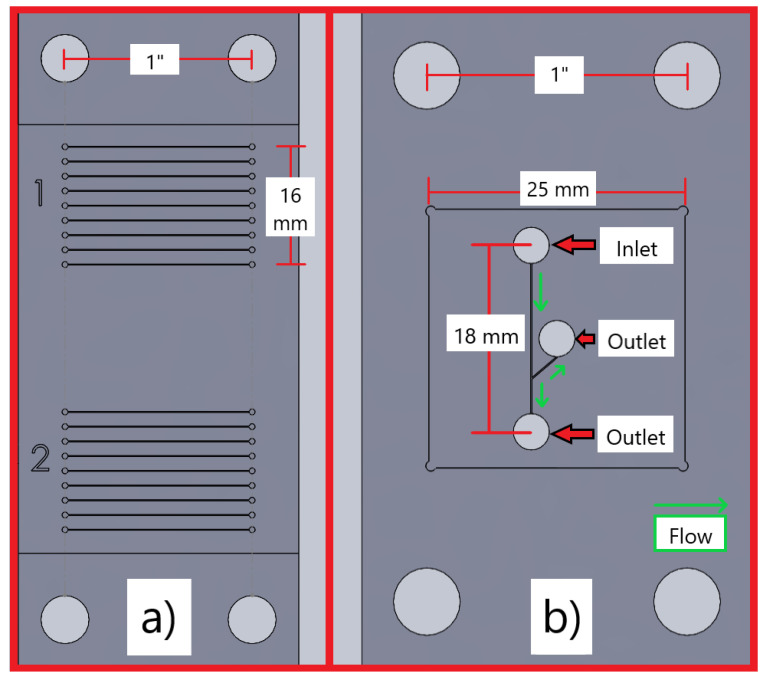
Two diagrams of microfluidic chip designs used in this research project. Microfluidic chip (**a**) consists of microchannels, which are 1′′ long and spaced 2 mm apart in two separate blocks of 9, each representing a replicate. The channels are 125 µm wide and 50 µm deep. The microfluidic chip (**b**) has one inlet port at the top and two outlet ports; it is designed for sorting microparticles. The channels are 125 µm wide and 100 µm deep.

In Figure 2a, all microchannels are 125 µm wide, 1′′ (25.4 mm) long, and 50 µm deep. They are milled into a 200 µm deep pocket, to ensure a consistent depth of cut. Each of the nine configurations between the feed rate and speed in Table 1 is cut into a block of channels in Figure 2a. A separate chip is made to test both the wet and dry milling processes. These values in Table 1 are derived from the previous research [25].

A particle-sorting microfluidic chip design is presented in Figure 2b. All the channels are 125 µm wide and 100 µm deep, milled into a 200 µm deep pocket, which is designed for a 25 mm × 25 mm piece of PMMA to enclose the channel. There is a single inlet at the top of the chip, an outlet at the bottom of the chip, and a side channel in the middle for assisting in particle manipulation.

### 2.3. Micro-Milling Fabrication

**Figure 3:**
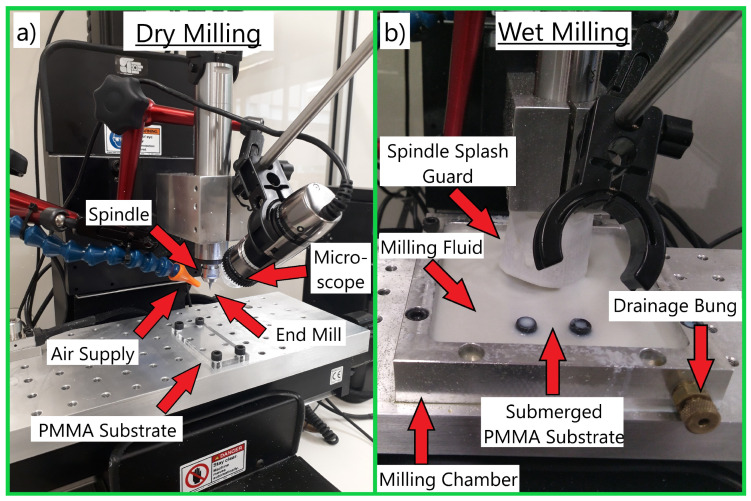
The two different milling machine configurations used in experimentation. Image (**a**) demonstrates the dry milling process, where the air is blown into the microchannels being cut directly into the PMMA substrate to clear debris away from the end mill attached to the spindle, improving the surface quality. Image (**b**) demonstrates a modified chamber for milling fluid to submerge the substrate, allowing debris to lift away from the end mill.

There are two main configurations for operating the micro mill. In the dry milling process, an air supply blows pressurised air into the microchannel, clearing debris from the milling area; Figure 3a. A camera is positioned so the milling coordinates can be set using visual feedback. An alternative configuration is the PMMA substrate being submerged in a milling fluid within a custom-made self-contained milling chamber to improve surface quality; Figure 3b [29]. The end mill cuts underneath the fluid, allowing for the debris to be lifted away from the milling area, improving the surface quality. The spindle is shrouded in a splash guard to prevent excessive quantities of fluid from spilling. The milling fluid is made from a concentrate of <60% distillates (petroleum), <20% alkanes C14-16 chloro-10, <5% monoethanolamine, <3% triazine triethanol, <3% triethanolamine, and is diluted 20:1, which is a commonly used mixture in standard CNC (computer numerical control) machining [32]. The microchannels were milled with a 100 µm end mill (100M2X300S, Performance Micro Tool, Janesville, WI, USA) with a Minitech micro-milling machine (Mini-Mill/3, Minitech, Norcross, GA, USA). A depth-of-cut of approximately 1/5 of the tool diameter is adhered to, as during the experimentation, it was the maximum value that could be used without increasing the risk of tool breakage [25].

### 2.4. Solvent Bonding

**Figure 4:**
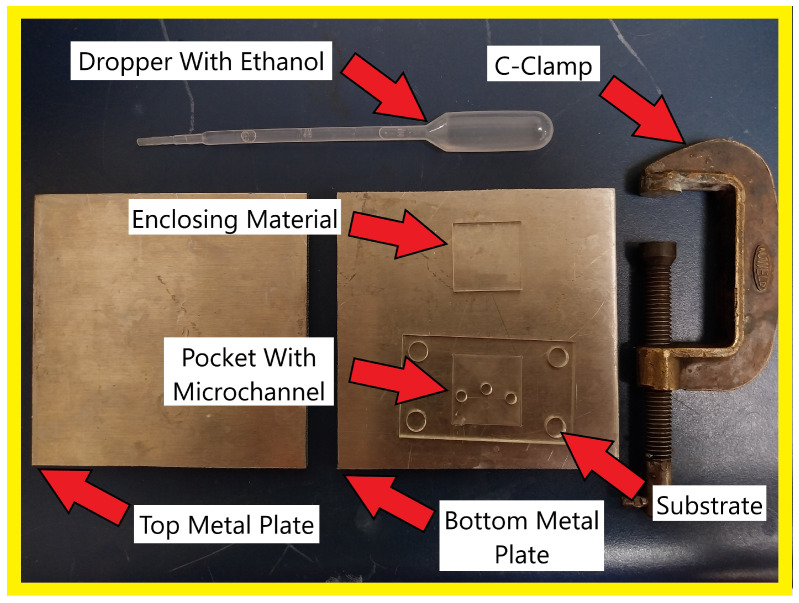
How solvent bonding is conducted with the equipment in the image. An unenclosed microfluidic chip has ethanol applied to the pocket, enclosing material is placed into the same pocket. Sandwiched between two aluminium metal plates, clamped together using a C-Clamp. This is put into the oven at 90 degrees Celsius for up to 12.5 min.

The conventional method for bonding PMMA to PMMA is using acetone and pressure to create a permanent seal [33,34]. This successfully bonded the acrylic together, but once smaller microchannels (such as the ones in this article) are used, they often collapse during the bonding process, leaving the channel closed off, and rendering the microfluidic chip unusable. This prompted research into different kinds of solvents. In one study, a mixture of 20% 1,2-dichloroethane and 80% ethanol and pressure was used at room temperature [35]. However, this process was ineffective at forming a bond, which could contain fluid without leakage in our experimentation. Another study used a substrate soaked in ethanol and baked in the oven for 30 min [36]; however, it still damaged microchannels. Through experimentation, this method was modified and the solvent was only applied to the pocket; the chip was then enclosed, sandwiched between aluminium plates, and baked at 90 degrees Celsius for up to 12.5 min. This resulted in a bond strong enough to seal without damaging the microfluidic chip as shown in Figure 4.

### 2.5. Attachment System

There are multiple approaches to interfacing with microfluidic chips. Some designs use wells to passively let the fluid run through the microfluidic chip; however, it is insufficient for precise manipulation. Modular designs use prefabricated fittings, e.g., twist and lock, which are too complex to rapidly fabricate, leaving integrated systems as the best choice [37]. The use of glue is not desirable as it makes the connection permanent and can degrade over time. Therefore, a press-fit mechanism was considered optimal for the rapid prototyping in this project [37,38].

**Figure 5:**
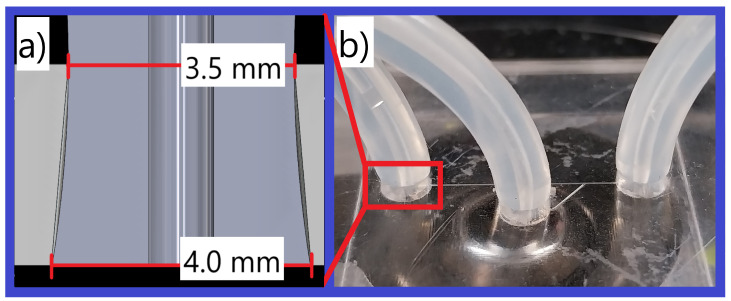
This figure highlights the implementation of the microfluidic attachment system used in experiments. Image (**a**) shows a cross-section of the attachment system used to connect microfluidic tubing to the microfluidic chip. It uses 4 mm of medical tubing and it compresses it to 3.5 mm, causing it to become stuck to the microfluidic chip. Image (**b**) shows the prototype of Image (**a**).

A simple low-cost microfluidic attachment system was designed to allow for easy removal and re-attachment when conducting experiments. This method is demonstrated in Figure 5a; the 4 mm wide medical tube is compressed through a 3.5 mm constriction that expands toward the bottom of the well. This causes a rubber pressure force to hold the tube, allowing fluid to be injected at pressure without leakage. With no fluid in the tube, it requires 2.33 N of perpendicular tensile force or 242 kPa of gauge pressure across 34.48 mm^2^. This is likely the minimum value, as an increase in fluid pressure will lead to a corresponding increase in the force required to dislodge it. The attachment system is presented in Figure 5b.

### 2.6. Surface Profiling

There are two primary methods used to profile the surface of an object: contact profileometery (CP) and non-contact profileometery (NCP). NCP involves the use of a laser and a receptor to measure the vertical distance by reflection off the surface. CP utilises a physical probe to measure the vertical displacement as it travels along the surface [39]. NCP failed to provide valid data due to the transparency of the PMMA interfering with the reflection of the laser. Therefore, CP was conducted via a stylus profilometer (Dektak, Bruker, Billerica, MA, USA) to measure the surface of the microchannels. Profilometry was conducted on every microchannel produced in Figure 2a, using the parameters in Table 1, on both replicates. The measurement was repeated three times at the top, middle, and bottom of each individual channel.

The data produced from these measurements are presented as distance values from 0 µm to 1000 µm and a vertical displacement set from an assigned zero-value determined by the profilometer. A linear trend was applied across each data batch to level off the slope. Measurement errors due to dust in the microchannels were removed from the dataset. The vertical displacement is converted to the difference from the trend and all the values have the root mean square applied to determine the roughness value for each test. The three results for each channel are averaged to calculate the overall roughness of the channel and the standard deviation of that roughness, determining the parameters’ effect on the roughness of the microchannels.

### 2.7. Physical Testing

**Figure 6:**
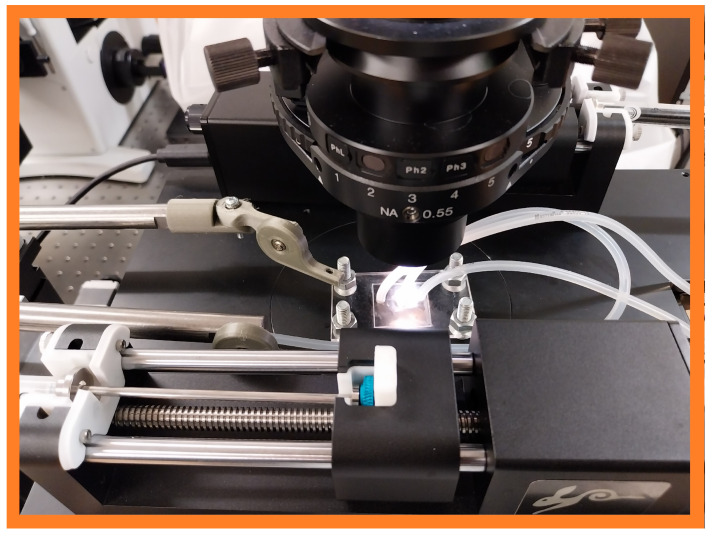
The experimental sorting chip is demonstrated with one inlet (on the **left**) and two outlets (on the **middle** and **right**). It is placed on a microscope stage so that the inside of the channel can be viewed with the microscope camera and feet are added to each corner to hold the microfluidic chip in place under observation.

Physical testing is used to validate that functional microfluidic chips can be fabricated using the methodologies developed in this research. The channels were fabricated with either the wet milling process in Figure 3a or the dry milling process in Figure 3b. This parameter had the greatest effect on surface quality in preliminary experimentation, which will allow for stronger differences in collected observations. An example of an experimental setup is provided in Figure 6. Particles were mixed into Milli-Q (purified) water and the mixture was injected into the microfluidic chip design; Figure 2b. Detected particles are characterised through image processing, selecting the particle with the greatest area. Its horizontal velocity is tracked, quantifying how particle interactions with the surfaces of the microchannel affect particle motion. This will be repeated three times in each channel on the same particle and repeated on both rough and smooth microchannel surfaces. Each particle’s velocity will be increased to over 20 µm/s (if possible) using a micro-pump pumping water into the channel at 0.1 µL/s. Once this velocity is reached, the pump will be switched off, allowing the particle to decelerate down to a complete stop.

### 2.8. Statistical Methods and Data Analysis

Results from profilometry were recorded into CSV files. While profilometry was conducted, the probe was observed under the microscope and contact points with dust were noted in the CSV file. These files were merged into a single workbook, and a linear trend was fitted on each measurement to level off the surface. At data points where the probe contacted dust (indicated by a significantly larger spike, which was noted as dust in the data sheets), the values at these distances were adjusted to match the trend values. The root-mean-square error from the trendline was calculated, representing the average surface roughness within a section of the microchannel. The overall average roughness for the entire microchannel was determined by averaging the measurements from the top, middle, and bottom sections, and the standard deviation was also calculated.

The averages were organised into a single spreadsheet and a full factor DOE (design-of-experiment) was conducted to determine the significance of each parameter and interaction using linear models. The average surface roughness was calculated for each parameter (as the interactions were insignificant) to generate the main effects plot which demonstrated the configuration of parameters had the smoothest surface.

## 3. Results and Discussion

### 3.1. Channel Appearance

**Figure 7:**
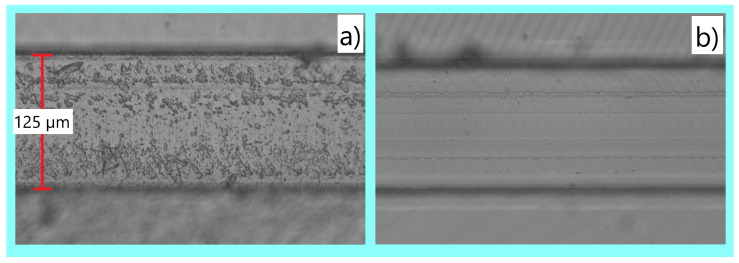
This figure directly compares two different microchannels: (**a**) demonstrates the rough appearance of a poorly manufactured microchannel with an average sampled surface roughness of 96 nm; (**b**) demonstrates the smooth appearance of a well-fabricated channel with a surface roughness of 24.9 nm.

When viewing a microfluidic channel it is apparent when the surface quality is significantly rougher, as showcased in Figure 7. In Figure 7a, there are many visible scars on the surface of the microchannel, corresponding to a significantly higher surface roughness value of 96 nm. Figure 7b is much smoother with less pronounced concentric milling features, resulting in a much lower surface roughness value of 24.9 nm specific to that image.

### 3.2. Profilometry Data

**Figure 8:**
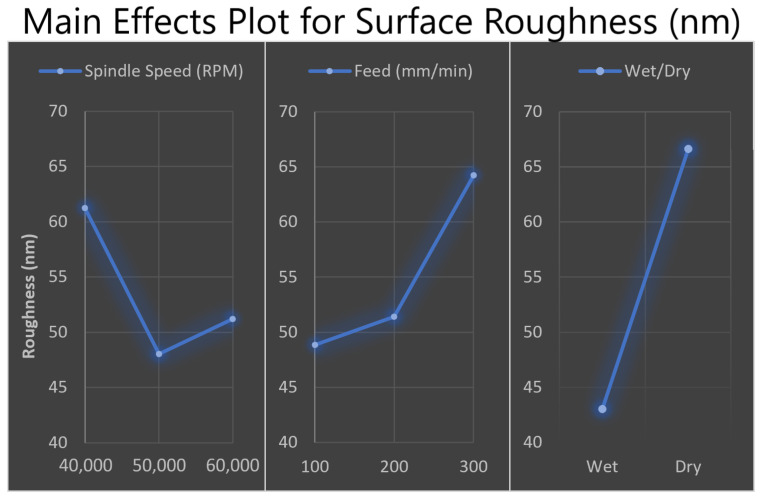
The main effects plot demonstrates which combination of factors is most effective at minimising surface roughness (50,000 RPM at 100 mm/min using the wet milling process).

During testing, a moderate quantity of dust infiltrated the microchannels. In some instances of measuring, it could not be avoided, so data from affected sections of the measurement were removed to improve the accuracy of results. The data in Figure 8 show the superiority of the wet milling process over dry milling, with the largest improvement in average roughness decreasing from 66 nm to 43 nm. Additionally, using the lowest feed rate of 100 mm/min and a spindle speed of 50,000 RPM, wet milling achieved the best average results of 30.2 nm across both replicates.

By comparing the achieved surface roughness with the literature in Table 2, it is apparent that the surface quality created using our specific process is high. The competing result has a surface roughness of 24 nm [28], just 6 nm less than what was achieved in the experiments. It is difficult to determine what milling parameters are ideal as different micro-milling machines will have different optimal parameters based on the technical specifications of that machine and the tooling used. A key point of difference in the process of this research involves the use of a self-contained milling chamber, which fully immerses the substrate in a made-for-purpose milling fluid. Applying a single drop of water [28] is not substantial enough for large milling operations as there is a significant chance of it running dry and long-term exposure of machinery to plain water can cause corrosion, affecting the performance and longevity of micro-milling machines. Additionally, narrower channels (125 µm wide as opposed to 200 µm wide) with a smaller 100 µm tool (as opposed to a 200 µm tool), demonstrate a separate area of skill [28].

### 3.3. Physical Experimentation

**Figure 9:**
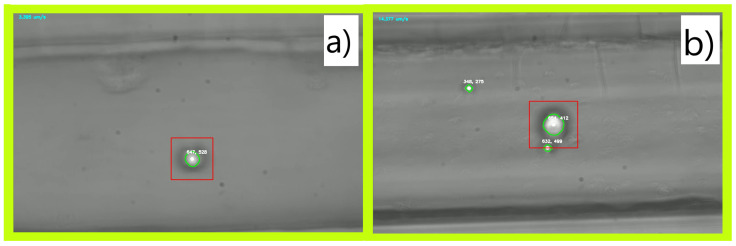
Two images of microchannels under a microscope being directly compared; (**a**) shows the particle tracked for all the data gathered from the rough microchannel (the particle is 10.0 µm in diameter); (**b**) shows the particle tracked for all the data gathered from the smooth microchannel (the particle is 13.2 µm in diameter).

**Figure 10:**
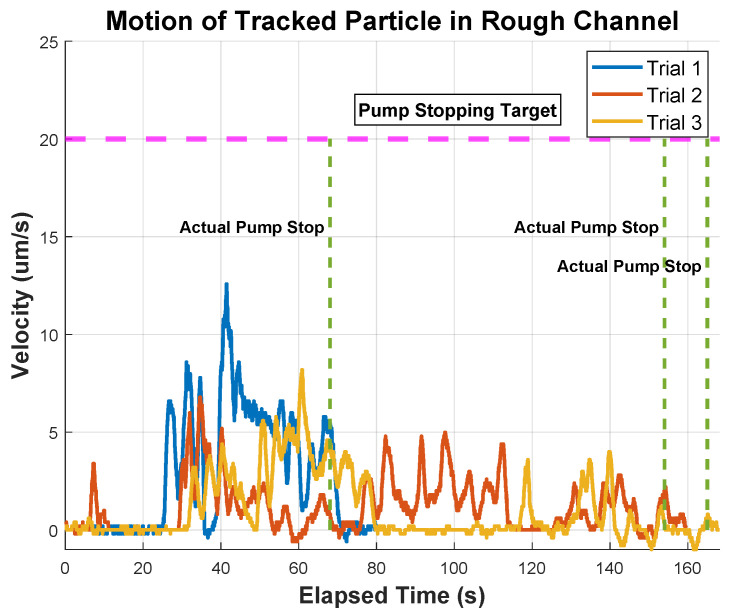
A diagram depicting the motion of the same microparticle travelling down a rough microchannel on a microfluidic chip. The particle is pushed down the channel with a flow of 0.1 µL/s and exhibits an erratic motion. Since the particle did not reach the target of 20 µm/s, it was stopped when it reached the end of the channel.

**Figure 11:**
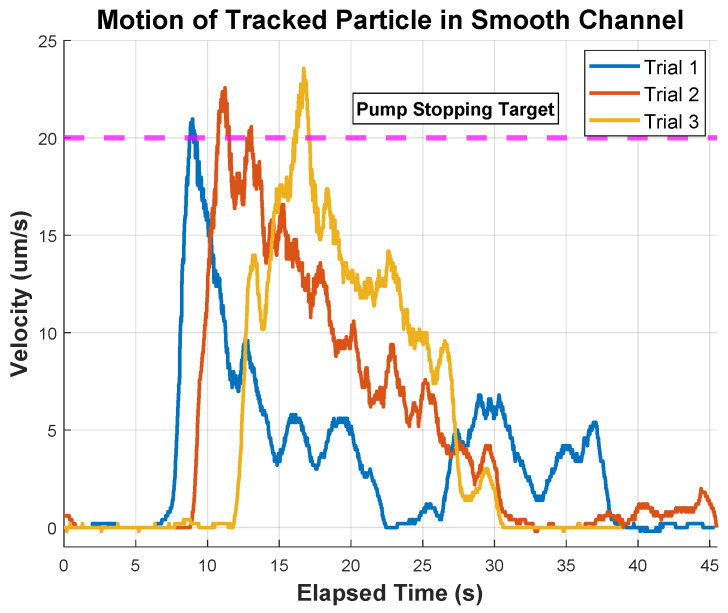
Diagram depicting the motion of the same microparticle travelling down a smooth microchannel on a microfluidic chip. The particle is pushed down the channel with a flow of 0.1 µL/s and exhibits a consistent pattern of movement with less friction slowing it down. The pump was stopped when the velocity went above 20 µm/s.

The tracked particles in this experiment are highlighted in Figure 9a,b. The velocity of particles in the rough microchannel is presented in Figure 10, demonstrating an erratic movement pattern. The particle randomly starts and stops and takes different amounts of time to traverse the microchannel, ranging from 76 s to over 160 s. In all trials, the particle was incapable of reaching the target velocity of 20 µm/s. Alternatively, in Figure 11, a consistent pattern emerges. All the trials can reach the same target velocity of 20 µm/s in a mostly smooth acceleration curve, and the pattern of deceleration is consistent, however, it does take a longer time (than the rough channel) to come to a complete stop (likely due to the lower frictional resistance). The three lines of the graph have a more similar shape than the rough data, indicating a higher level of consistency in motion and controllability. Additionally, the microchannel traversal time has been reduced to a range of 39 s to 46 s, showing an overall improvement in the efficiency of transit.

The rough and smooth channel tests yielded significantly different velocity–time graphs for particles travelling in a microfluidic chip. In the rough channel, a peak velocity was never achieved as the pump was switched off (at a significantly later time) to prevent the particle from accelerating out of frame. In the rough channel, particles could not exceed a velocity of 13 µm/s across all trials. One reason for this limitation is that increased surface roughness reduces the actual flow in the channel by increasing friction against the fluid [40]. This corresponds to an increase in noise in measured velocities, influencing the functionality of micro-dispensers and affecting small-scale fabrication processes. It also affects the injection of chemicals into micro-reactors, altering chemical production and reducing the efficiency of micro-engines, which affects the desired end result in a broad range of scientific applications [41]. Additionally, increased surface roughness can cause further deviations from the surface mean, trapping particles and temporarily hindering their movement.

### 3.4. Impact

The complete fabrication process developed in this research has the potential to provide a rapid alternative to conventional microfluidic chip fabrication techniques. When contrasted with other micro-milling processes in Table 2, the presented method can produce consistently high-quality microfluidic chips in a method suitable for higher production volumes. During experimentation, fully functional microfluidic chips were produced in approximately an hour, allowing for testing to be conducted on the same day a chip was fabricated. Adoption of this process into the research and development cycle could help produce results in a much quicker time frame, for a lower cost than conventional methodologies.

## 4. Conclusions

A novel wet micro-milling process was successfully used to rapidly fabricate fully functional microfluidic devices using low-cost materials. The surface profile of the microchannels was analysed using contact profileometery to measure the optimal surface roughness of 30.4 nm, demonstrating the effectiveness of our fabrication process and which milling parameters are best for the machine we use. Moreover, an easy-to-operate microfluidic system was developed using removable tubing and syringe pumps, which successfully manipulated micro-particles, demonstrating the competitiveness of our new process, which reduced the minimum transit time from 76 s down to 39 s. In the future, it would be valuable to test the method developed in this research on a range of materials, showcasing the versatility of our method. The findings in this research have the potential to help advance microfluidic technology and increase the accessibility of microfluidics for all researchers in the fields of medicine, biology, chemistry, ecology, and aerospace [42].

## Figures and Tables

**Table 1:** Milling parameters and values used in surface quality experiment. This table shows the translational speed of the end mill (feed rate), the micro-milling configuration (wet/dry), and the rotational speed of the end mill (speed) at different parameter levels.

Parameters	Values		
Feed rate (mm/min)	100	200	300
Wet/Dry	Wet	Dry	
Speed (kRPM)	40	50	60

**Table 2:** Optimal milling parameters from different literature studies and our study.

-	Study				
Parameters	Ref [21]	Ref [26]	Ref [27]	Ref [28]	This
Tool Size (µm)	127	200	450	200	100
Feed rate (mm/min)	25	300	300	10	100
Depth of Cut (µm)	N/A	10	50	10	20
Speed (kRPM)	5	20	150	4	50
Roughness (nm)	420	130	38	24	30

## Data Availability

The original contributions presented in the study are included in the article/Supplementary Material, further inquiries can be directed to the corresponding author.

## Supplementary Materials

The following supporting information can be downloaded at: https://www.mdpi.com/article/10.3390/mi15070905/s1, Figure S1: Appendix S1—Different Milling Widths and Tool Sizes; Text S2: Appendix S2—Particle Tracking and Velocity; Table S3: Appendix S3—Profilometry Data Sheet; Figure S4: Appendix S4—PMMA Technical Data Sheet.
