# Supplementary material for: An Alternative Micro-Milling Fabrication Process for Rapid and Low-Cost Microfluidics
Source: Micromachines (Basel). 2024 Jul 11;15(7):905. doi: 10.3390/mi15070905 (PMC11279306; doi:10.3390/mi15070905)
Supplement: Supplementary file 1 [file micromachines-15-00905-s001.zip › micromachines-3080253-supplementary.pdf]

Supplementary Materials

Appendix S1 – Different Milling Widths & Tool Sizes

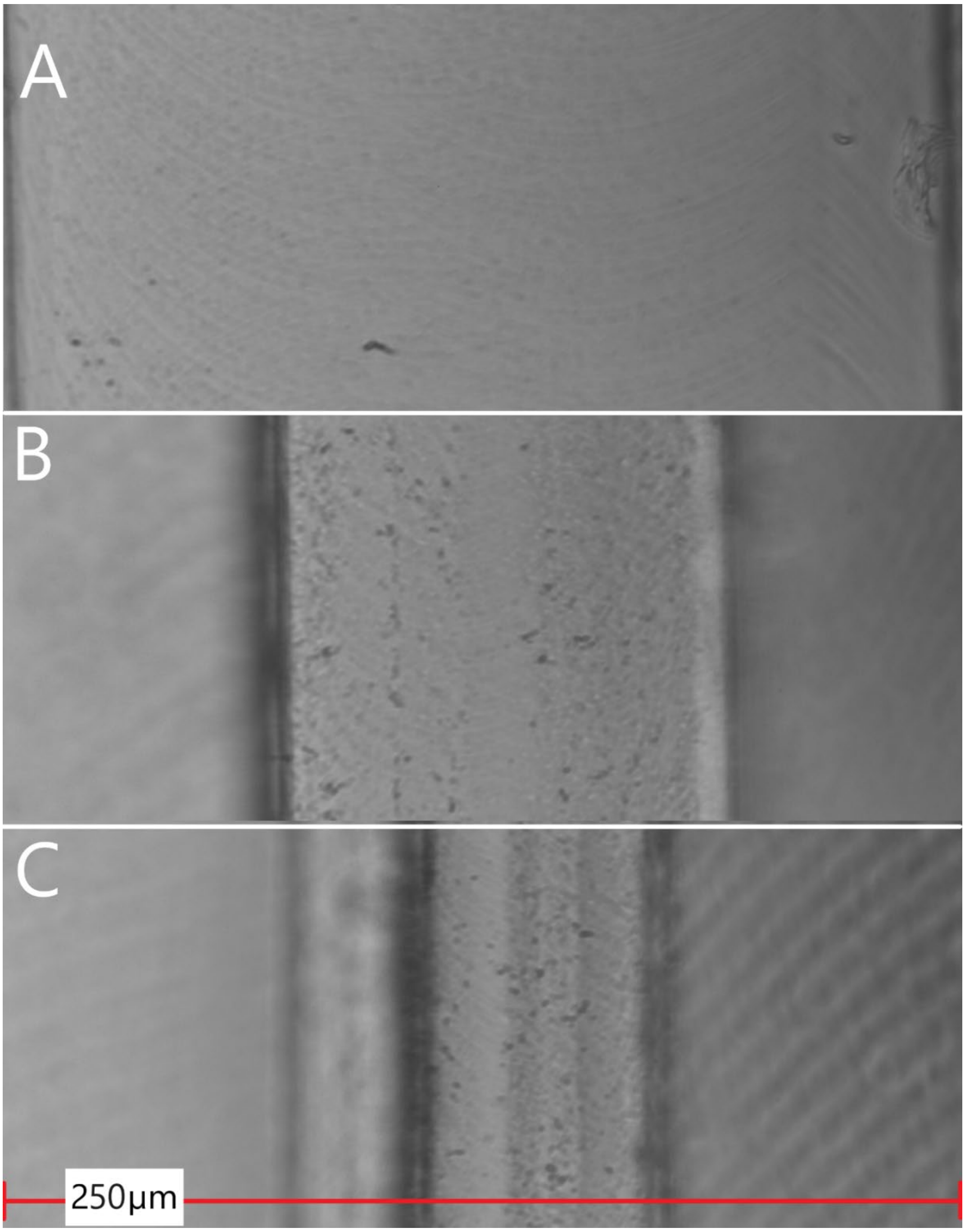

To demonstrate possibility of creating different size channels, an example above of a 250-um wide, 125 um wide, and a 62.5 um wide microfluidic channels created with the wet milling process at 60000 RPM at 100 mm/min feed-rate. Created with 200 um, 100 um and 50 um tools respectively.

Appendix S2 – Particle Tracking & Velocity

The code used for tracking the particles is listed below. It isolates the particles from each frame of the camera while it is opened on a display and positioned within the bounding box. The area is calculated, and the largest particle is tracked is to prevent the tracked particle from changing or latching on to false positives. The current position and previous position in the x-axis are determined allowing the difference to be calculated which is converted to micrometres by multiplying it with the ratio between the width of the channel and the number of pixels in it in the frame (125/626) giving the distance in micrometres. The camera is locked to 30 fps and a queue list of the last 30 calculated speeds is filled and summed to determine how far the particle travelled in the last second, giving a speed in um/s. This information is saved with its current positional information and time stamp into a .txt file.

$$Pixel\ Movement = Current\ Position - Previous\ Position$$

$$Distance\ (\mu m) = Pixel\ Movement * \frac{125}{626}$$

$$Speed = \sum_{i=1}^{30} Distannce\ (\mu m)_i$$

```
import numpy as np
import cv2
from mss import mss
from PIL import Image
import imutils
import math
import time
import csv

bounding_box = {'top': 110, 'left': 0, 'width': 1300, 'height': 850}

sct = mss()
kernel = cv2.getStructuringElement(cv2.MORPH_ELLIPSE, (3, 3))
largest = [0, 0, 0]
fourcc = cv2.VideoWriter_fourcc(*'mp4v')
out = cv2.VideoWriter('output.mp4', fourcc, 30.0, (1300, 850))
speeds = []
Positions = []

while True:
    sct_img = sct.grab(bounding_box)
    img = np.array(sct_img)

    output = img.copy()
    output[:] = 0
```

```python
    blur = cv2.GaussianBlur(img,(9,9),1)
    grayscale = cv2.cvtColor(blur, cv2.COLOR_BGR2GRAY)

    mask1 = cv2.inRange(grayscale, 0, 170)
    mask1 = cv2.bitwise_not(mask1)
    mask1 = cv2.dilate(mask1, kernel, iterations=10)

    image = cv2.bitwise_and(mask1, grayscale)

    # find contours in the binary image
    cnts = cv2.findContours(mask1.copy(), cv2.RETR_EXTERNAL,
        cv2.CHAIN_APPROX_SIMPLE)
    cnts = imutils.grab_contours(cnts)
    xsum = 0
    prev_x = largest[1]
    largest = [0 , 0, 0]
    for c in cnts:
        # compute the center of the contour
        M = cv2.moments(c)
        cX = int(M["m10"] / M["m00"])
        cY = int(M["m01"] / M["m00"])
        # draw the contour and center of the shape on the image
        cv2.drawContours(img, [c], -1, (0, 255, 0), 2)
        cv2.circle(img, (cX, cY), 7, (255, 255, 255), -1)
        cv2.putText(img, str(cX)+ ", " + str(cY), (cX - 20, cY - 20),
            cv2.FONT_HERSHEY_SIMPLEX, 0.5, (255, 255, 255), 2)
        xsum = xsum + cX
        curarea= cv2.contourArea(c)
        if curarea > largest[0]:
            largest = [curarea, cX, cY]

    if largest[0] > 0:

        if len(speeds) > 30:
            speeds.pop(0)

        speeds.append((largest[1]-prev_x)*125/626)
        speed = sum(speeds)
        speed = round(speed, 3)
        Positions.append([round(time.time()-1684564000, 2), speed])

        cv2.putText(img, str(speed) + (" um/s"), (20,20),
cv2.FONT_HERSHEY_SIMPLEX, 0.5, (0, 255, 255), 2)

        radius = math.sqrt(largest[0] / math.pi) + 50

        x = int(largest[1]-radius)
```

```
        y = int(largest[2]-radius)

        a = int(largest[1]+radius)
        b = int(largest[2]+radius)

        cv2.rectangle(img, (x, y), (a, b), (255,0,0), 2)

    img = cv2.cvtColor(img, cv2.COLOR_BGR2RGB)

    cv2.imshow("output", img)
    out.write(img)

    if (cv2.waitKey(1) & 0xFF) == ord('q'):
        cv2.destroyAllWindows()
        break

file = open('Position.txt','w')

for pos in Positions:
    file.write(', '.join(str(x) for x in pos) + "\n")
file.close()

out.release()
cv2.destroyAllWindows()
```

Appendix S3 – Profilometry Data Sheet

The test chips were produced on separate days in Replicate 1 and Replicate 2

Replicate	Wet/Dry	Spindle Speed (RPM)	Feed Rate (mm/min)	Roughness (nm)	STD DEV (nm)	STD Order
1	Dry	40000	100	96.0	36.5	7

1	Dry	40000	200	72.3	3.0	36
1	Dry	40000	300	86.8	2.7	23
1	Dry	50000	100	64.6	15.8	28
1	Dry	50000	200	56.0	5.9	30
1	Dry	50000	300	60.9	5.4	11
1	Dry	60000	100	58.7	15.6	15
1	Dry	60000	200	74.6	11.4	5
1	Dry	60000	300	86.9	11.0	16
1	Wet	40000	100	31.7	5.1	34
1	Wet	40000	200	67.6	46.8	35
1	Wet	40000	300	66.5	30.0	6
1	Wet	50000	100	30.9	14.5	2
1	Wet	50000	200	26.6	2.7	8
1	Wet	50000	300	52.1	9.2	22
1	Wet	60000	100	47.7	1.9	24
1	Wet	60000	200	37.7	13.9	20
1	Wet	60000	300	57.6	3.9	31
2	Dry	40000	100	63.4	14.4	27
2	Dry	40000	200	59.5	5.6	4
2	Dry	40000	300	90.4	11.2	14
2	Dry	50000	100	50.9	10.9	9
2	Dry	50000	200	62.3	22.2	17
2	Dry	50000	300	49.9	4.8	26
2	Dry	60000	100	54.1	12.6	32
2	Dry	60000	200	51.1	7.0	18
2	Dry	60000	300	61.3	3.0	33
2	Wet	40000	100	24.9	9.5	29
2	Wet	40000	200	34.7	7.4	10
2	Wet	40000	300	41.4	4.7	12
2	Wet	50000	100	29.5	17.3	13
2	Wet	50000	200	32.9	8.2	19
2	Wet	50000	300	60.0	17.0	21
2	Wet	60000	100	34.0	5.6	25
2	Wet	60000	200	41.4	9.9	3
2	Wet	60000	300	57.3	8.8	1

Replicate 1 average = 59.7 nm with a standard deviation of 19.9 nm

Replicate 2 average = 49.9 nm with a standard deviation of 13.0 nm

Appendix S4 - PMMA Technical Data Sheet

Technical Data Sheet

ECO CAST

ASTARIGLAS® ECO CAST

General Properties

	Test Method	Units	ASTARIGLAS® ECO CAST
Relative Density	ISO 1183	g/mc³	1.19
Water Absorption	ISO 62	%	0.35

Mechanical Properties

	Test Method	Units	ASTARIGLAS® ECO CAST
Tensile Strength @23°C	ISO 527	MPa	69
Tensile Strength, Rupture	ISO 527	MPa	68
Elongation at Break @23°C	ISO 527	%	4.2
Flexural Strength	ISO 178	MPa	116
Modulus of Elasticity	ASTM D638	psi	460,000
Flexural Modulus	ISO 178	MPa	3300
Impact Strength - Charpy-Unnotched	ISO 179 / I fu	$kJ.m^{-2}$	12.5
Izod Impact Strength, Notched	ISO 180/ I A	$kJ.m^{-2}$	1.35
Shear Strength	ASTM D732	psi	8,900
Shear Modulus	ASTM D732	psi	167,500
Rockwell Hardness	ISO 2039-2	M scale	100

Thermal Properties

	Test Method	Units	ASTARIGLAS® ECO CAST
Vicat Softening Point	ISO 306 A	°C	>110
Heat Deflection Temperature	ISO 75	°C	95
Hot Forming Temperature	N/A	°C	140 - 180
Maximum Continuous Service Temperature	N/A	°C	80 - 85
Coefficient of Thermal - Conductivity	ASTM C177	BTU /(hr) (sgft) (°F/in)	1.3
Coefficient of Thermal - Expansion-Linear	ASTM D696	$x\ 10^{-5}.\ K^{-1}$	7
Flammability UL94 HB	UL94		HB
Flammability (burning rate on 0.236")	ASTM D635	in/min	0.99
Self-Ignition Temperature	ASTM D1929	°F	874
Smoke Density Rating (on 0.236")	ASTM 2843	%	9.9

Optical Properties

	Test Method	Units	ASTARIGLAS® ECO CAST
Light Transmission	ASTM D1003	% (3 mm)	>92
Haze	ASTM D1003	% (3 mm)	0.55
Refractive Index	ISO 489 A		1.49

Electrical Properties

	Test Method	Units	ASTARIGLAS® ECO CAST
Surface Resistivity	IEC 93	Ω	$>10^{14}$
Volume Resistivity	ASTM D 257	Ohm-cm	1.6×10^{16}
Electrical Strength	IEC 243	kV/mm	30

Note:
The standards value quoted are not always strictly equivalent and based on tests on representatives samples. The information given in this publication is based on our general experience and given in good faith. It is intended as a general guide and must not be considered as a binding specification. No warranty is given or is to be implied. In no way does this information incurs the liability of Astari Niagara Internasional, especially in infringement of the rights of a third party.
